# Construction and validation of artificial intelligence pathomics models for predicting pathological staging in colorectal cancer: Using multimodal data and clinical variables

**DOI:** 10.1002/cam4.6947

**Published:** 2024-03-28

**Authors:** Yang Tan, Run Liu, Jia‐wen Xue, Zhenbo Feng

**Affiliations:** ^1^ Department of Pathology The First Affiliated Hospital of Guangxi Medical University Nanning Guangxi China

**Keywords:** artificial intelligence, colorectal cancer, pathological staging, pathomics, nomogram

## Abstract

**Objective:**

This retrospective observational study aims to develop and validate artificial intelligence (AI) pathomics models based on pathological Hematoxylin–Eosin (HE) slides and pathological immunohistochemistry (Ki67) slides for predicting the pathological staging of colorectal cancer. The goal is to enable AI‐assisted accurate pathological staging, supporting healthcare professionals in making efficient and precise staging assessments.

**Methods:**

This study included a total of 267 colorectal cancer patients (training cohort: *n* = 213; testing cohort: *n* = 54). Logistic regression algorithms were used to construct the models. The HE image features were used to build the HE model, the Ki67 image features were used for the Ki67 model, and the combined model included features from both the HE and Ki67 images, as well as tumor markers (CEA, CA724, CA125, and CA242). The predictive results of the HE model, Ki67 model, and tumor markers were visualized through a nomogram. The models were evaluated using ROC curve analysis, and their clinical value was estimated using decision curve analysis (DCA).

**Results:**

A total of 260 deep learning features were extracted from HE or Ki67 images. The AUC for the HE model and Ki67 model in the training cohort was 0.885 and 0.890, and in the testing cohort, it was 0.703 and 0.767, respectively. The combined model and nomogram in the training cohort had AUC values of 0.907 and 0.926, and in the testing cohort, they had AUC values of 0.814 and 0.817. In clinical DCA, the net benefit of the Ki67 model was superior to the HE model. The combined model and nomogram showed significantly higher net benefits compared to the individual HE model or Ki67 model.

**Conclusion:**

The combined model and nomogram, which integrate pathomics multi‐modal data and clinical‐pathological variables, demonstrated superior performance in distinguishing between Stage I–II and Stage III colorectal cancer. This provides valuable support for clinical decision‐making and may improve treatment strategies and patient prognosis. Furthermore, the use of immunohistochemistry (Ki67) slides for pathomics modeling outperformed HE slide, offering new insights for future pathomics research.

## 1 INTRODUCTION

Colorectal cancer is one of the common digestive tract tumors, ranking third globally among malignant tumors and second in cancer‐related deaths.^1^ The American Joint Committee on Cancer (AJCC) TNM system for colorectal cancer staging is the optimal guide for treatment decisions.^2^

The pathological TNM staging of colorectal cancer is predominantly based on the assessment of tumor infiltration depth, lymph node invasion, and the existence of metastasis to distant sites, encompassing Stage I, Stage II, Stage III, and Stage IV^3^ The diagnosis and prognosis of colorectal cancer patients are closely related to precise staging. Staging‐related colorectal cancer prognosis studies have shown an inverse correlation between stage and prognosis, meaning that Stage I–II has a better prognosis than Stage III–IV.^2^, ^4^, ^5^, ^6^ For patients with Stage I–II colorectal cancer, the most effective treatment is surgery, often without the need for adjuvant or neoadjuvant therapy.

Colonoscopy is among the most prevalent cancer screening methods and proves to be highly effective in reducing the mortality rate of colorectal cancer.^7^ However, the frequent utilization of colonoscopy results in the identification of a higher number of colorectal cancer patients. In essence, this means that an increased number of colorectal cancer specimens necessitate a more extensive involvement of pathologists in delivering precise diagnoses and staging evaluations. Nevertheless, pathological specimen diagnosis depends heavily on the rich personal experience and solid theoretical knowledge of pathologists. Variability among individuals can easily lead to differences between observers.^8^ As a result, the challenges of pathological staging are amplified because many patients are already in the advanced stage when diagnosed, as early‐stage cancer often presents no obvious symptoms.

In recent years, the rapid development of artificial intelligence (AI) and machine learning technologies has provided new opportunities to address this problem. AI is a hot research area in the field of pathological diagnosis. Currently, AI models in colorectal cancer mainly focus on gland segmentation, tumor classification, tumor microenvironment characterization, and prognosis prediction.^7^, ^9^, ^10^, ^11^, ^12^

Hüneburg and other researchers utilized AI for real‐time application in Lynch syndrome patients, revealing that AI‐assisted colonoscopy optimizes endoscopic surveillance in lynch syndrome patients, notably enhancing the detection of flat adenomas.^13^ In a large‐scale study conducted in asymptomatic populations in China, Xu et al. compared AI‐assisted colonoscopy with traditional methods, indicating a substantial improvement in overall adenoma detection rates with AI assistance.^14^ Similarly, Wallace et al. conducted a multicenter study in Western countries investigating AI's impact on the miss rate of colorectal adenomas, demonstrating a halved miss rate with AI‐assisted detection.^15^ These findings underscore the clear role of AI in screening and detecting colorectal adenomatous polyps, highlighting AI‐assisted colonoscopy as a highly promising approach. However, leveraging immune scoring in predicting prognosis in colorectal cancer faces challenges, restricting its application in prognostication. Foersch et al. developed a multistain deep learning model for predicting prognosis and treatment response in rectal cancer, exhibiting robust predictive performance.^16^ Additionally, Yang et al. attempted to quantify immune scoring using AI, distinguishing between different risk profiles in stage II colorectal cancer patients.^17^ These studies suggest that AI holds promise in offering valuable insights into the tumor microenvironment of colorectal cancer. Furthermore, in prognostic research related to colorectal cancer, Susič and colleagues employed machine learning algorithms to predict 1–5 year survival rates in colorectal cancer patients, attempting to identify crucial variables influencing patient survival.^18^ Wang et al. established a nomogram model based on multi‐omics features including pathological, radiological, and immunological characteristics for predicting prognosis of colorectal cancer lung metastasis, demonstrating favorable clinical utility.^19^ By directly predicting diagnosis, treatment effectiveness, and prognosis from pathological slides, healthcare can be more precisely implemented, making it more efficient.^20^

Research on AI in colorectal cancer staging has primarily focused on predicting early colorectal cancer invasion depth using colonoscopy images and predicting lymph node metastasis using pathological tissue images.^21^, ^22^, ^23^ The former is associated with the T stage, and the latter with the N stage. However, few researchers have conducted studies using machine learning to predict colorectal cancer TNM staging based on colonoscopy or pathological tissue images.

Therefore, this study aims to develop and validate AI‐based pathomics models using pathological tissue slide images to predict the pathological staging of colorectal cancer, distinguishing between Stages I–II and III. We aim to provide a powerful tool for physicians to more accurately assess the condition of colorectal cancer patients, offering valuable information for individualized treatment and patient management. This research is expected to provide crucial references and guidance for future colorectal cancer treatment and pathology studies.

## 2 MATERIALS AND METHODS

### 2.1 Patient population

Our retrospective study was approved by our institutional ethics review board. Inclusion criteria for this study were as follows: (1) Colorectal cancer pathological tissue slides stored in the form of digital pathology slide scans (whole slide images, WSI), including hematoxylin–eosin (HE) stained slides and immunohistochemical (IHC) slides (Ki67 marker); (2) clear pathological staging; (3) no history of preoperative chemoradiation; (4) no metastasis. Exclusion criteria included: (1) Blurriness in some or all regions of the pathological digital slide; (2) absence of cancer tissue in the pathological digital slide. This study ultimately included data from 267 patients who underwent curative surgery for colorectal cancer in our hospital, with a random 80/20 split into training (213 patients) and testing (54 patients) cohorts.

### 2.2 Image acquisition and annotation

Pathological tissue slide scanning was performed using a slide scanner provided by Shenzhen Shengqiang Technology Co., Ltd. (http://www.sqray.com/product/list), at 20× resolution. The obtained digital pathology images were in SDPC format and were converted to SVS format. Subsequently, the tumor regions in the pathological WSIs were annotated using QuPath software.^24^ QuPath is an open‐source software specialized for digital pathology image analysis (version 0.4.3, https://qupath.github.io/), used to annotate tumor regions in pathological WSIs.^25^ These annotated WSIs were then divided into 512 × 512‐sized patches.^24^ The same process was applied to both HE and Ki67 slides.

### 2.3 The steps and workflow for model construction

**FIGURE 1:**
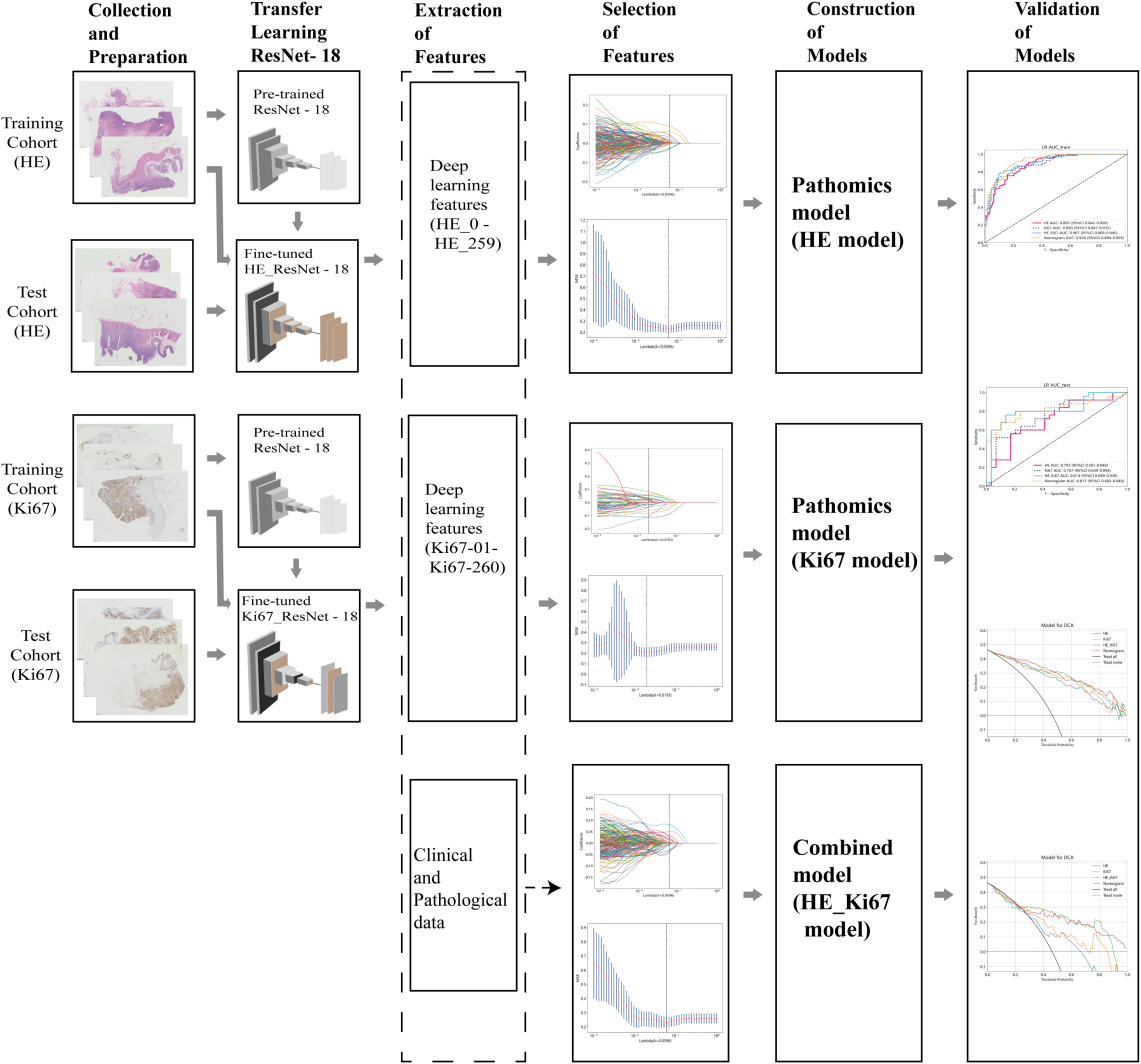
The workflow of pathomics HE model, pathomics Ki67 model, and combined model in this study.

The model construction process involves the following steps: (1) Collection and preparation of pathological slides and clinical data; (2) tumor annotation of pathological slides and transfer learning with deep learning models; (3) extraction and selection of deep features from pathological slides; (4) division of the dataset into training and testing sets; (5) construction of the pathomics logistic regression (LR) models; (6) validation of the pathomics models. The workflow of the pathomics model in this study is presented in Figure 1.

### 2.4 Extraction and selection of pathological slide features

The completed patches were subjected to transfer learning on a ResNet‐18 model, pre‐trained on the ImageNet dataset, to create a deep learning model for recognizing tumor and non‐tumor areas in patches. Model training utilized stochastic gradient descent as the optimization algorithm to adjust the weights and biases of the deep learning model, further minimizing the cross‐entropy loss function. We used the cross‐entropy loss function to train the model to better predict sample labels. The training was carried out with a learning rate of 0.01 and implemented with an adaptive moment estimation optimizer for three epochs with a batch size of 128. After training, the penultimate layer of the fine‐tuned deep model was utilized for feature extraction.

The extracted pathological deep features (from both HE and Ki67 slides) were subjected to several operations. First, each feature was standardized by z‐score normalization (mean of 0 and standard deviation of 1), making the data conform to a standard normal distribution. Then, Spearman's rank correlation coefficient, a statistic measuring the correlation between two variables, was employed to assess the correlations among features. When the Spearman correlation coefficient between two features was greater than 0.9, one of the features was retained. Finally, feature dimension reduction was performed using L1‐regularized least absolute shrinkage and selection operator (LASSO) regression, which selected the most relevant features and generated a sparse model, meaning that only a few features significantly contributed to the prediction outcome, enhancing the model's interpretability and generalization. The selected features were used for constructing the pathological models (HE model or Ki67 model). Furthermore, clinical‐pathological variables with univariate regression analysis *p*‐values <0.05 were integrated with deep learning features from HE slides and Ki67 slides. Subsequently, feature selection was applied using the method described above. The selected features were then utilized to construct the combined model (HE_Ki67 model).

### 2.5 Development and validation of models

In this study, machine learning using LR was employed to construct pathological models (HE model and Ki67 model) and the HE_Ki67 model using the selected features, with colorectal cancer Stage I–II and Stage III as binary outcome variables. The predictive results of the HE model based on HE slides and the Ki67 model based on Ki67 slides were separately used as features, along with clinical‐pathological variables with *p*‐values <0.05 from univariate regression analysis, to construct a nomogram. Once models' construction were completed, validation was performed in the testing cohort. Model performance was assessed based on metrics such as the area under the receiver operating characteristic curve (ROC AUC), accuracy, sensitivity, specificity, positive predictive value (PPV), and negative predictive value (NPV). Subsequently, decision curve analysis (DCA) was conducted to evaluate the clinical utility of the models, reflecting the net benefit at different threshold probabilities in the training and external validation cohorts.

### 2.6 Statistical analysis

Clinical baseline features were subjected to t‐tests, chi‐square tests, or Fisher's exact tests using SPSS software (version 25.0, IBM). T‐tests were used for continuous variables with homogeneity of variances, presented as x ± s. Chi‐square tests or Fisher's exact tests were used for categorical variables, presented as ratios. A two‐tailed *p*‐value <0.05 indicated statistical significance. Python software (version 3.7.17; http://www.python.org) was used for Spearman rank correlation tests, z‐score normalization, LASSO regression analysis, univariate regression analysis, and multivariate regression analysis, as well as for generating ROC curves and clinical decision curves. Variables with a univariate regression analysis *p*‐value <0.05 were included in the multivariate regression analysis, and these variables were also incorporated into the combined model and nomogram construction. The hardware environment for this study includes an Intel Core i7‐13700KF 3.40GHz CPU, an NVIDIA GeForce RTX 4070Ti GPU with CUDA 12.2.79, and 64GB DDR4 memory. The software environment comprises Python 3.7.12 as the programming language, scikit‐learn 1.0.2 as the machine learning library, and Jupyter Notebook 6.5.4 as the development environment.

## 3 RESULTS

### 3.1 Clinical and pathological features of patients

This study involved 267 patients diagnosed with colorectal cancer, comprising 213 patients in the training cohort and 54 patients in the testing cohort. Summary statistics of clinical and pathological features of the patients are presented in Table 1. Statistical analysis was performed for Stages I–II and Stage III in both the training and testing cohorts. Age, gender, tumor size, differentiation, vascular thrombosis, neural invasion, CK20 (IHC), HER2 (IHC), and most hematologic tumor markers showed no significant statistical differences. CA242 and CA199 displayed a statistical significance with a *p*‐value less than 0.05 in the training cohort.

**TABLE 1:** Clinical and pathological characteristics in the training and test cohorts.

Characteristics	Training cohort (*n* = 213)			Test cohort (*n* = 54)		
	I–II Stage, (*n* = 114)	III Stage, (*n* = 99)	*p*‐value	I–II Stage, (*n* = 29)	III Stage, (*n* = 25)	*p*‐value
Age (years), *n* (%)						
≤60	55 (48.25)	51 (51.52)	0.735	12 (41.38)	7 (28.00)	0.459
>60	59 (51.75)	48 (48.48)		17 (58.62)	18 (72.00)	
Gender, *n* (%)						
Female	39 (34.21)	43 (43.43)	0.215	11 (37.93)	8 (32.00)	0.866
Male	75 (65.79)	56 (56.57)		18 (62.07)	17 (68.00)	
Tumor size (cm), mean ± SD	4.52 ± 1.97	4.19 ± 1.68	0.392	4.44 ± 1.55	4.65 ± 1.90	0.951
Differentiation degree, *n* (%)						
Well differentiation	14 (12.28)	13 (13.13)	0.569	4 (13.79)	6 (24.00)	0.099
Moderate differentiation, *n* (%)	90 (78.95)	81 (81.82)		18 (62.07)	18 (72.00)	
Poor differentiation	10 (8.77)	5 (5.05)		7 (24.14)	1 (4.00)	
Vascular thrombus, *n* (%)						
Absent	72 (63.16)	64 (64.65)	0.934	10 (34.48)	16 (64.00)	0.059
Present	42 (36.84)	35 (35.35)		19 (65.52)	9 (36.00)	
Nerve invasion, *n* (%)						
Absent	69 (60.53)	66 (66.67)	0.432	18 (62.07)	18 (72.00)	0.629
Present	45 (39.47)	33 (33.33)		11 (37.93)	7 (28.00)	
CK20 (IHC), *n* (%)						
Negative	2 (1.75)	None	0.211	None	1 (4.00)	0.352
Focal positive	16 (14.04)	9 (9.09)		5 (17.24)	2 (8.00)	
Positive	96 (84.21)	90 (90.91)		24 (82.76)	22 (88.00)	
HER2 (IHC), *n* (%)						
0	39 (34.21)	35 (35.35)	0.951	13 (44.83)	9 (36.00)	0.628
1+	48 (42.11)	38 (38.38)		10 (34.48)	8 (32.00)	
2+	24 (21.05)	23 (23.23)		6 (20.69)	7 (28.00)	
3+	3 (2.63)	3 (3.03)		None	1 (4.00)	
CA242 (U/mL), mean ± SD	11.39 ± 18.16	24.04 ± 42.15	0.008	9.98 ± 8.69	21.02 ± 36.96	0.670
CA724 (U/mL), mean ± SD	2.66 ± 3.65	4.72 ± 9.30	0.303	3.97 ± 5.90	4.67 ± 9.82	0.821
AFP (ng/mL), mean ± SD	2.66 ± 1.19	3.29 ± 4.03	0.628	2.39 ± 1.20	2.72 ± 1.68	0.445
CEA (ng/mL), mean ± SD	8.80 ± 16.46	14.12 ± 26.25	0.057	4.32 ± 4.41	22.80 ± 72.62	0.099
CA125 (U/mL), mean ± SD	17.12 ± 24.40	21.84 ± 27.36	0.295	14.40 ± 11.23	36.04 ± 71.83	0.849
CA153 (U/mL), mean ± SD	10.71 ± 5.11	11.35 ± 7.12	0.831	10.00 ± 4.95	8.70 ± 3.74	0.386
CA199 (U/mL), mean ± SD	24.42 ± 104.77	48.30 ± 135.01	0.018	8.65 ± 6.60	386.73 ± 1774.84	0.097

*Note*: CK20 (IHC), the presence of cytokeratin 20 in tissue samples by using of immunohistochemistry. HER2 (IHC), the presence of Human Epidermal Growth Factor Receptor 2 in tissue samples by using of immunohistochemistry. HER2 (IHC) results are categorized as follows: 0, no HER2 expression; 1+, weakly positive HER2 expression; 2+, moderately positive HER2 expression; 3+, strongly positive HER2 expression.

Abbreviation: AFP, alpha‐fetoprotein; CA125, carbohydrate antigen125; CA153, carbohydrate antigen153; CA199, carbohydrate antigen 199; CA242, carbohydrate antigen 242; CA724, carbohydrate antigen 724; CEA, carcinoembryonic antigen; IHC, immunohistochemistry; SD, standard deviation.

Univariate regression analysis of the 15 variables in the clinical and pathological data revealed *p*‐values less than 0.05 for CA242, CA724, CEA, and CA125. A subsequent multivariate regression analysis of the variables with *p*‐values less than 0.05 showed that CA242 and CA125 remained significant. The odds ratios (OR) and *p*‐values for univariate and multivariate analysis of clinical and pathological variables are shown in Table 2.

**TABLE 2:** Univariate and multivariate logistic regression analysis of clinical‐pathological variables associated with colorectal cancer staging.

Variables	Univariate analysis		Multivariate analysis	
	OR (95% CI)	*p*‐value	OR (95% CI)	*p*‐value
Age	1.001 (0.904–1.107)	0.990		
Gender	0.937 (0.845–1.040)	0.302		
Tumor size (cm)	0.984 (0.957–1.011)	0.330		
Differentiation degree	0.896 (0.806–0.997)	0.091		
Vascular thrombus	0.928 (0.837–1.028)	0.233		
Nerve invasion	0.928 (0.835–1.031)	0.243		
CK20 (IHC)	1.128 (0.988–1.288)	0.135		
HER2 (IHC)	1.023 (0.963–1.088)	0.533		
CA242 (U/mL)	1.003 (1.002–1.005)	0.001	1.003 (1.001–1.004)	0.005
CA724 (U/mL)	1.009 (1.002–1.016)	0.041	1.007 (1.000–1.014)	0.114
AFP (ng/mL)	1.020 (1.001–1.040)	0.081		
CEA (ng/mL)	1.002 (1.001–1.004)	0.027	1.002 (1.000–1.004)	0.068
CA125 (U/mL)	1.002 (1.000–1.004)	0.040	1.002 (1.000–1.003)	0.036
CA153 (U/mL)	1.002 (0.993–1.010)	0.729		
CA199 (U/mL)	1.000 (1.000–1.000)	0.161		

*Note*: For detailed explanations of the abbreviations for the variables, please refer to Table 1.

Abbreviation: AFP, alpha‐fetoprotein; CA125, carbohydrate antigen125; CA153, carbohydrate antigen153; CA199, carbohydrate antigen 199; CA242, carbohydrate antigen 242; CA724, carbohydrate antigen 724; CEA, carcinoembryonic antigen; IHC, immunohistochemistry; SD, standard deviation.

### 3.2 Establishment of deep learning models for identifying tumor regions and extraction of features

**FIGURE 2:**
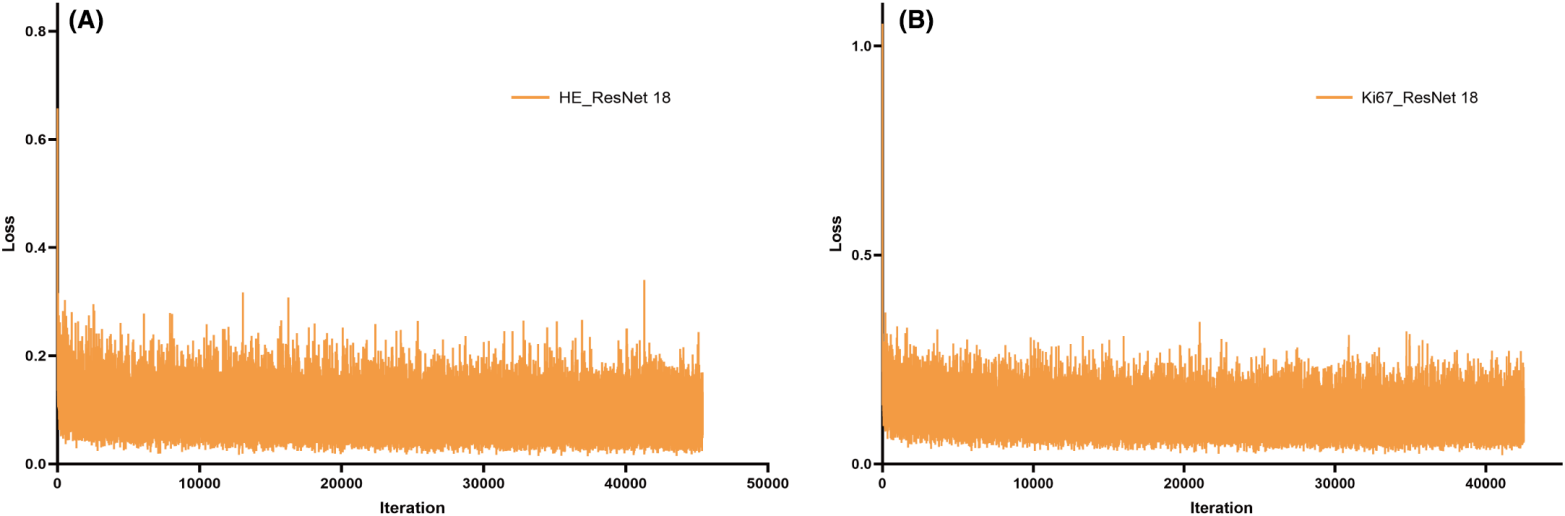
The loss value of deep learning HE_ResNet‐18 model (A) and Ki67_ResNet‐18 model (B) in the training patches with the iteration steps.

HE_ResNet‐18 and Ki67_ResNet‐18 models were created by utilizing the patches from pathological HE slides and Ki67 slides after transfer learning with ResNet‐18. The loss values of the deep learning HE_ResNet‐18 model and Ki67_ResNet‐18 model during training patches with iteration steps can be seen in Figure 2. These deep learning models were primarily employed for identifying tumor and non‐tumor regions. The performance evaluation of the deep learning models is presented in Table 3. The penultimate layer of HE_ResNet‐18 and Ki67_ResNet‐18 models was used to extract features from patches, respectively. A total of 260 deep learning features were obtained from pathological HE and Ki67 images, labeled as HE_0 to HE_259 and Ki67‐01 to Ki‐260.

**TABLE 3:** The performance evaluation of the deep learning HE_ResNet‐18 model and Ki67_ResNet‐18 model.

Model	Cohort	Label	AUC (95% CI)	Accuracy	Sensitivity	Specificity	PPV	NPV	Precision	Recall	F1	Threshold
HE_ResNet‐18	Training	Non‐tumor	0.992 (0.9915–0.9918)	0.954	0.951	0.967	0.993	0.807	0.993	0.951	0.971	0.831
		Tumor	0.992 (0.9915–0.9918)	0.954	0.967	0.951	0.807	0.993	0.807	0.967	0.879	0.170
	Test	Non‐tumor	0.99 (0.9903–0.9907)	0.952	0.950	0.964	0.992	0.800	0.992	0.950	0.971	0.864
		Tumor	0.99 (0.9903–0.9907)	0.952	0.963	0.950	0.800	0.992	0.800	0.963	0.874	0.137
Ki67_ResNet‐18	Training	Non‐tumor	0.986 (0.9860–0.9864)	0.943	0.949	0.942	0.784	0.988	0.784	0.949	0.858	0.186
		Tumor	0.986 (0.9860–0.9864)	0.943	0.942	0.949	0.988	0.784	0.988	0.942	0.964	0.815
	Test	Non‐tumor	0.987 (0.9869–0.9874)	0.947	0.946	0.947	0.795	0.988	0.795	0.946	0.864	0.203
		Tumor	0.987 (0.9869–0.9874)	0.947	0.947	0.947	0.988	0.795	0.988	0.947	0.967	0.798

### 3.3 Construction and performance of pathomics models and combined model

**FIGURE 3:**
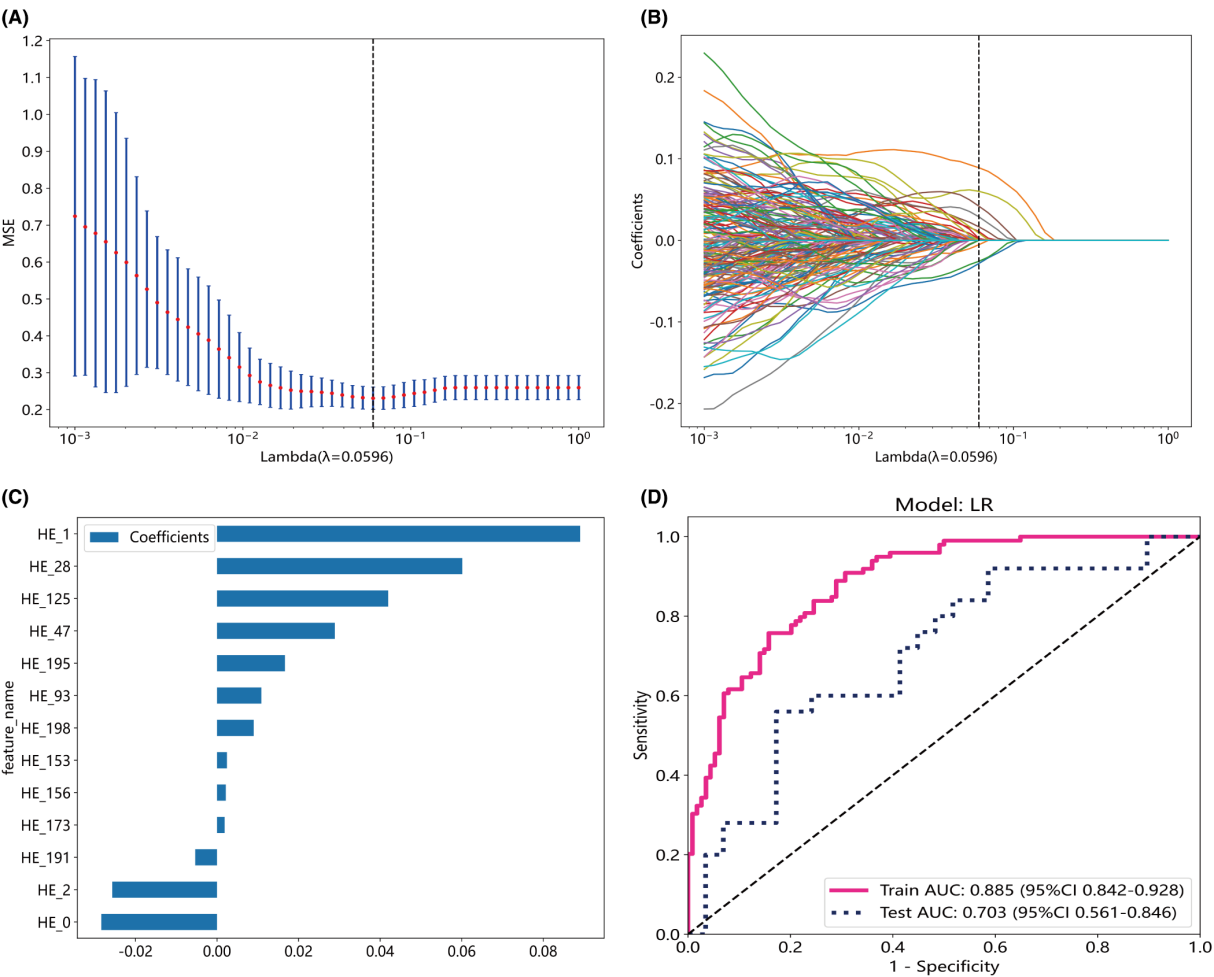
Feature selection from whole‐slide images of pathological Hematoxylin–Eosin (HE) stained sections and the performance of the pathomics HE model constructed with selected features. Optimal λ values are chosen based on 10‐fold cross‐validation and minimum mean squared error (MSE), represented by vertical dashed lines (A). Display least absolute shrinkage and selection operator (LASSO) coefficients for different λ values, where vertical dashed lines indicate the number of features corresponding to the optimal λ value (B). Following the application of LASSO regression for feature selection, exclusively those features exhibiting non‐zero coefficients were retained (C). The receiver operating characteristic curves of the pathomics HE model in both the training and test cohorts were showed (D).

**FIGURE 4:**
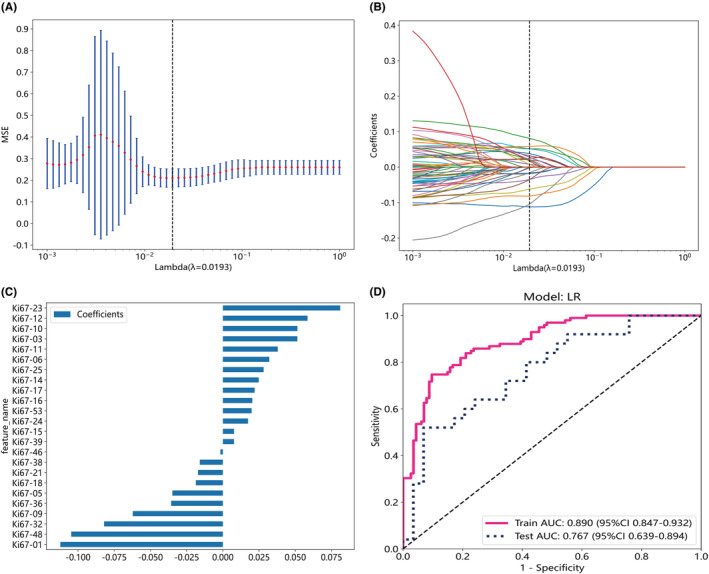
Feature selection from whole‐slide images of pathological immunohistochemistry (Ki67) stained sections and the assessment of the pathomics Ki67 model utilizing the chosen features. Optimal λ values were determined through 10‐fold cross‐validation and minimizing mean squared error (MSE), as depicted by vertical dashed lines (A). Visualization of least absolute shrinkage and selection operator (LASSO) coefficients for various λ values, with vertical dashed lines indicating the number of features corresponding to the optimal λ value (B). Following the application of LASSO regression for feature selection, only features with non‐zero coefficients were retained (C). Evaluation of receiver operating characteristic curves for the pathomics Ki67 model constructed in both the training and test cohorts were presented (D).

**FIGURE 5:**
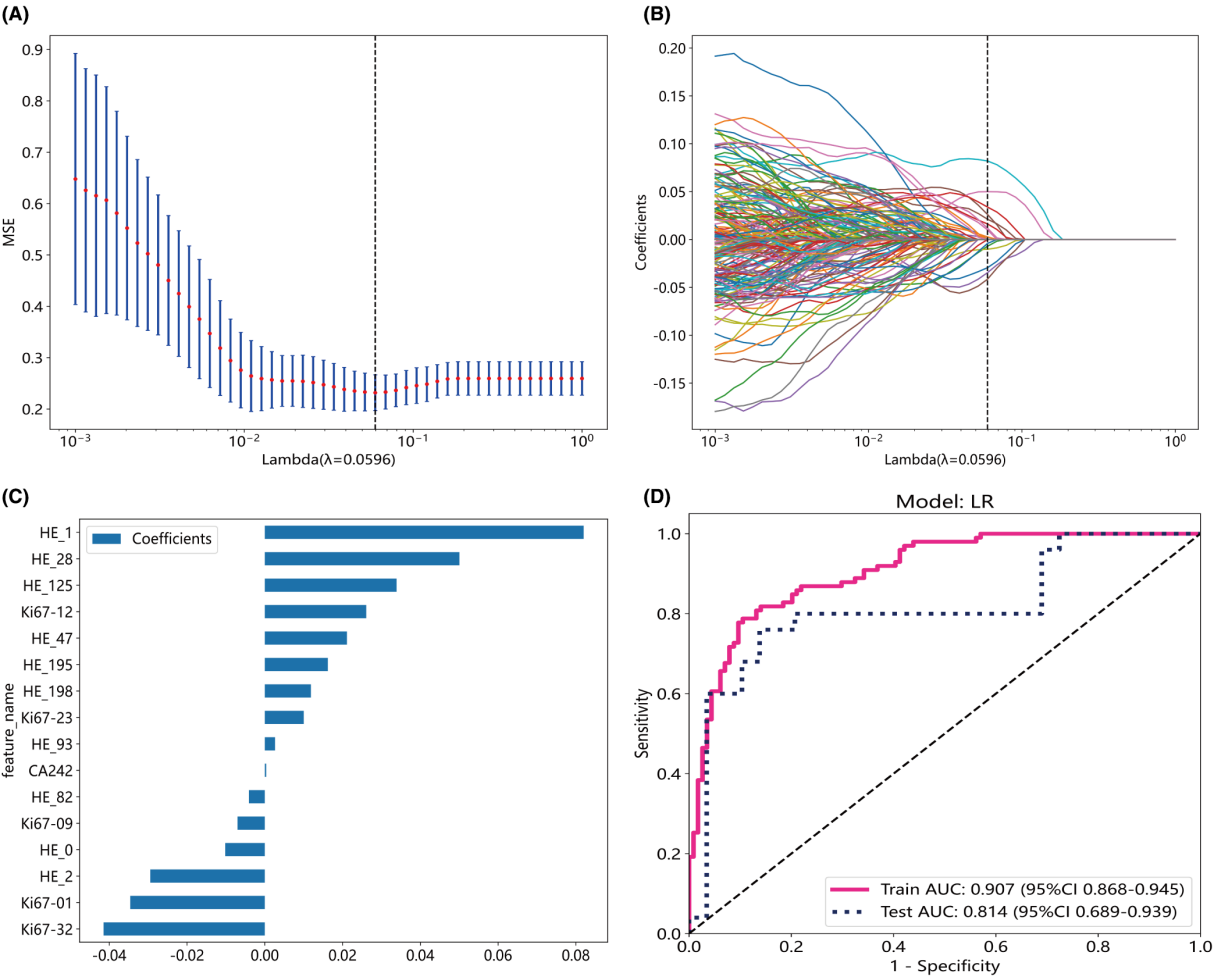
The integration of multimodal feature data from whole‐slide images of both HE and Ki67 sections with clinical variables for feature selection, and the evaluation of the combined (HE_Ki67) model's performance constructed with the selected features. Optimal λ values were determined through 10‐fold cross‐validation and minimizing mean squared error (MSE), as depicted by vertical dashed lines (A). Visualization of least absolute shrinkage and selection operator (LASSO) coefficients for various λ values, with vertical dashed lines indicating the number of features corresponding to the optimal λ value (B). Following the application of LASSO regression for feature selection, only features with non‐zero coefficients were retained (C). Presentation of receiver operating characteristic curves to assess the performance of the combined model in both the training and test cohorts (D).

The extracted feature data was normalized, and features with Spearman's correlation coefficient greater than 0.9 were reduced to one. The λ value corresponding to the minimum mean squared error (MSE) was selected (Figures 3A, 4A, and 5A), and a LASSO regression model was fitted based on the λ value (Figures 3B, 4B, and 5B). Feature dimension reduction was achieved by eliminating features with zero coefficients using LASSO regression. Following feature reduction, 13, 24, and 16 features were respectively selected (Figures 3C, 4C, and 5C). LR, a machine learning algorithm, was used to build the HE model, Ki67 model, and HE_Ki67 model using the selected features. The ROC curve results in the training and testing cohorts are shown in Figures 3D, 4D, and 5D. The ROC for HE model, Ki67 model, and HE_Ki67 model in the training cohort were 0.885 (95% CI 0.842–0.928), 0.890 (95% CI 0.847–0.932), and 0.907 (95% CI 0.868–0.945), while in the testing cohort, the ROC was 0.703 (95% CI 0.561–0.846), 0.767 (95% CI 0.639–0.894), and 0.814 (95% CI 0.689–0.939). Other performance parameters are displayed in Table 4.

**TABLE 4:** Performance of models for predicting discrimination between Stages I–II and Stage III colorectal cancer in training and test cohorts.

Model	Cohort	AUC (95% CI)	Accuracy	Sensitivity	Specificity	PPV	NPV	Precision	Recall	F1	Threshold
HE model	Training	0.885 (0.842–0.928)	0.793	0.693	0.909	0.898	0.720	0.898	0.693	0.782	0.661
	Test	0.703 (0.561–0.846)	0.704	0.828	0.560	0.686	0.737	0.686	0.828	0.750	0.186
Ki67 model	Training	0.890 (0.847–0.932)	0.831	0.904	0.747	0.805	0.871	0.805	0.904	0.851	0.419
	Test	0.767 (0.639–0.894)	0.741	0.931	0.520	0.692	0.867	0.692	0.931	0.794	0.245
Combined (HE_ki67)	Training	0.907 (0.868–0.945)	0.845	0.895	0.788	0.829	0.867	0.829	0.895	0.861	0.514
	Test	0.814 (0.689–0.939)	0.815	0.862	0.760	0.806	0.826	0.806	0.862	0.833	0.413
Nomogram	Training	0.926 (0.894–0.958)	0.850	0.833	0.869	0.880	0.819	0.880	0.833	0.856	0.590
	Test	0.817 (0.693–0.940)	0.796	0.897	0.680	0.765	0.850	0.765	0.897	0.825	0.526

### 3.4 Nomogram construction and models' comparison

**FIGURE 6:**
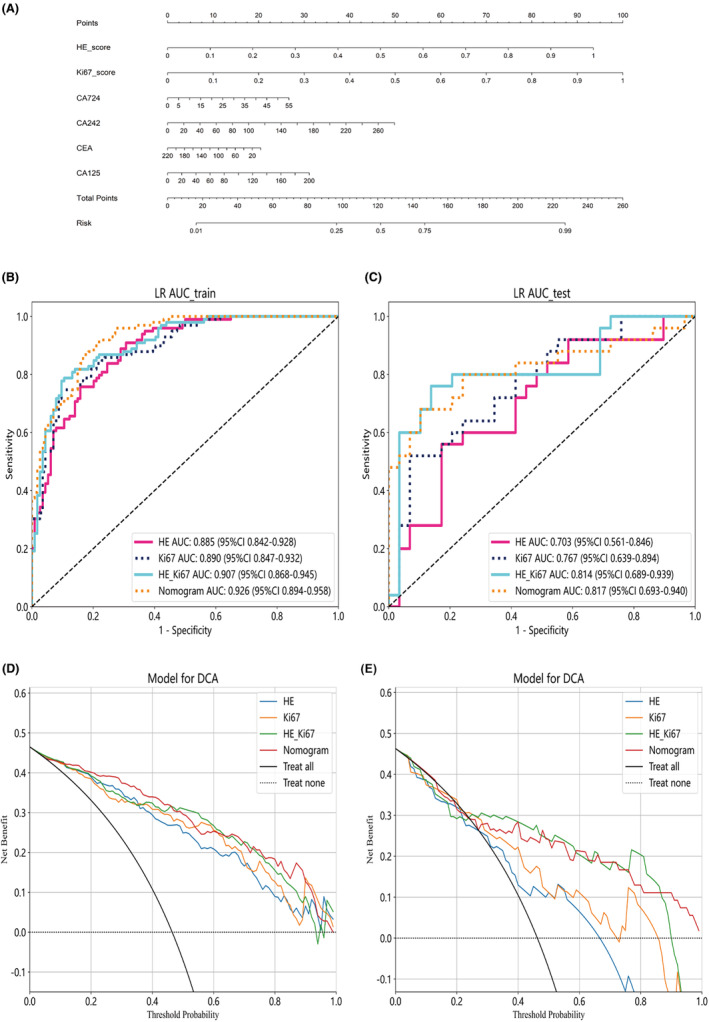
The construction of the nomogram was accomplished using the predictive outcomes of the pathomics HE model (HE_score) and pathomics Ki67 model (Ki67_score), in conjunction with clinical variables (CEA, CA724, CA125, and CA242) (A). ROC analysis of the pathomics HE model (HE), pathomics Ki67 model (Ki67), combined model (HE_Ki67) and nomogram was conducted in both the training (B) and test cohorts (C). Decision curve analysis (DCA) was performed on three models and nomogram for classifying between Stages I–II and Stage III colorectal cancer in the training (D) and test (E) cohorts, respectively.

In this study, the predictions from the HE model and Ki67 model were used as features for constructing a nomogram, along with clinical variables with a *p*‐value less than 0.05 from univariate regression analysis (Figure 6A). The ROC curve results in the training and testing cohorts are shown in Figure 6B,C. The AUC values in the training cohort were 0.926 (95% CI 0.894–0.958) and 0.817 (95% CI 0.693–0.940) in the testing cohort. Other performance parameters are presented in Table 4. DCA curves for the nomogram and other models are shown in Figure 6D,E. The results indicate that the nomogram based on HE medel, Ki67 model, and clinical variables, as well as the HE_Ki67 model, both demonstrate higher net benefits for classifying Stages I–II and Stage III colorectal cancer in both the training and testing cohorts compared to the HE model and Ki67 model. In the testing cohort, the HE_Ki67 model performs slightly better than the nomogram, and further data are needed to support this observation. Additionally, an interesting observation is that the Ki67 model's net benefit is higher than the HE model in both the training and testing cohorts.

## 4 DISCUSSION

In our study, multiple pathomics models were developed using the LR algorithm based on features extracted from pathological HE slides and Ki67 slides. The HE model was created using features extracted from HE slides, while the Ki67 model was developed using features from Ki67 slides. Furthermore, the combined model (HE_Ki7 model) was constructed based on clinical variables and the two aforementioned feature sets. Finally, the predictive outcomes of the HE model and Ki67 model, along with clinical variables, were incorporated into the construction of a nomogram. The results indicate that the combined model (HE_Ki7 model) and nomogram, leveraging features from multi‐modal data (HE and Ki67) along with clinical variables, outperform the single‐modal HE model or Ki67 model in identifying Stage I–II and Stage III colorectal cancer. In clinical DCA, both the combined model and nomogram demonstrate higher net benefits.

The pathological TNM staging of tumors is crucial for ensuring the appropriate selection of patients for treatment interventions because different stages require different treatment methods.^2^ Currently, the pathological TNM staging for colorectal cancer involves pathologists meticulously examining the tissue morphology of tumors and lymph nodes in postoperative curative specimens through a microscope, in combination with judgments based on other relevant clinical examinations. This analytical process consumes a significant amount of time in the work of pathologists. This is due to the need for comprehensive sampling of the tumor area and lymph nodes in curative specimens, which typically requires the preparation of dozens of wax blocks to complete. In other words, pathologists need to review dozens of pathological slides under the microscope to complete the pathological staging of colorectal cancer. Accurate pathological staging is inseparable from the experience and professional knowledge of pathologists, and variations among individuals can easily lead to differences in observations.^8^ The diagnostic variations in this regard and the relatively time‐consuming nature of completing colorectal cancer pathological staging underscore the urgent need for the application of AI in predicting colorectal cancer's pathological staging. This application can enhance diagnostic efficiency, accuracy, and consistency while also reducing healthcare costs for patients.

Research on the pathological staging of colorectal cancer using AI primarily focuses on the prediction of T‐stage related to the depth of invasion and N‐stage related to lymph node metastasis. Regarding depth of invasion, the use of deep learning on endoscopy images to predict the depth of intestinal wall invasion is a key area of research, particularly for early‐stage colorectal cancer (T1 stage). Deep learning models based on endoscopy images for predicting the depth of invasion in gastric cancer have shown better accuracy and specificity in AI predictions compared to real‐world endoscopy doctors.^26^ Furthermore, a deep learning‐based model for assessing colorectal cancer invasion, utilizing multimodal data, including endoscopy images, produced results on the validation set that were similar to those of endoscopy doctors,^21^ and even outperformed experienced endoscopy doctors.^27^ This suggests that the prospects for AI in predicting the depth of invasion in colorectal cancer are promising. However, the diagnostic accuracy still presents challenges,^28^ and further research data is needed for refinement and support. Currently, the prediction of colorectal cancer's T‐stage primarily relies on endoscopy images, and there are no AI models based on pathological tissue images for predicting T‐stage.

Regarding the prediction of lymph node metastasis in colorectal cancer, AI models are primarily built using imaging modalities such as CT/MRI and pathological HE‐stained whole‐slide images.^23^, ^29^, ^30^, ^31^, ^32^, ^33^ A meta‐review of AI models using CT/MRI to predict preoperative lymph node metastasis in colorectal cancer reported an AUC of 0.727.^30^ Studies on predicting lymph node metastasis in colorectal cancer based on pathological HE‐stained whole‐slide images have reported validation set AUC values ranging from 0.61 to 0.76.^29^, ^32^, ^33^ In a few cases, immunostained whole‐slide images have been used for predicting lymph node metastasis. One study utilized a random forest algorithm on immunohistochemical cytokeratin‐stained whole‐slide images to predict lymph node metastasis in early‐stage colorectal cancer.^22^ Another study, which used cytokeratin staining for predicting lymph node metastasis in pT1 stage colorectal cancer, did not show an advantage over HE staining.^34^ Further research is needed to confirm the value of pathological immunostained whole‐slide images in lymph node prediction.

The pathological staging of colorectal cancer involves a comprehensive analysis of tumor infiltration depth, lymph node status, and metastasis. Predicting T‐stage or N‐stage individually cannot provide a complete reflection of the overall pathological staging. Currently, there is a lack of machine learning research that simultaneously considers both T‐stage and N‐stage in the pathological staging of colorectal cancer. This study, to some extent, aims to fill this gap. In this study, for the first time, we used pathological tissue sections to establish AI models for predicting Stage I–II and Stage III colorectal cancer. Our results indicate that the Ki67 model developed using immunohistochemical labeling of whole‐slide images outperforms the HE model based on HE‐stained whole‐slide images in discriminating between Stage I–II and Stage III colorectal cancer. Whether in the training or validation datasets, the Ki67 model demonstrated higher AUC values and net benefits than the HE model (Figure 6B–E).

A significant feature of this study is the integration of pathological data with clinical variables. The combination of deep learning features extracted from HE and Ki67 whole‐slide images with clinical variables enhances the accuracy and robustness of the constructed combined model and nomogram. This is likely to provide a more comprehensive perspective on patient conditions. Interestingly, combining features from HE and Ki67 images with tumor markers (CEA, CA724, CA125, and CA242) in the combined model and nomogram results in better predictive performance. Impressive ROC AUC values were achieved in both the training and test datasets (Figure 6B,C), emphasizing the enhancement of predictive accuracy through the application of multimodal data. This finding underscores the importance of integrating different data sources to improve the accuracy of pathological staging, as different data types can capture complementary information. DCA was conducted in this study to assess the clinical utility of the models, reflecting their actual value at different probability thresholds. Importantly, the combined model and nomogram exhibited significantly higher net benefits compared to the HE and Ki67 models (Figure 6D, E). This suggests the potential of these models to assist diagnostic physicians in making more informed staging assessments.

In both univariate and multivariate analyses of this study, serum CA125 and CD242 were identified as independent risk factors. Previous research by scholars has shown an elevation in serum CA125 levels in advanced stages of primary colorectal cancer,^35^ and a positive correlation with tumor staging.^36^, ^37^ Simultaneously, CD242 has been associated with the invasion and staging of colorectal cancer.^38^ In the study conducted by Björkman et al., CA125 and CD242 emerged as significant prognostic factors for colorectal cancer, with levels increasing alongside higher tumor stages.^39^ Similar findings were reported by Luo et al., indicating a higher positivity rate for CA125 and CD242 in Stages III–IV compared to Stages I–II in colorectal cancer.^40^ In other words, there is a higher prevalence of elevated CA125 and CD242 values in Stages III–IV. The serum levels of CA125 and CD242 in this study exhibit a positive correlation with advanced stages of colorectal cancer, consistent with previous research.

Despite the encouraging results, it must be acknowledged that this study has limitations. Its retrospective nature and relatively small sample size may introduce biases and impact generalizability. Prospective studies on larger and more diverse cohorts are needed to further validate these models and their clinical utility. Additionally, this study focused on distinguishing Stage I–II and III colorectal cancer. Expanding the scope of research to include other stages and subtypes may provide a more comprehensive understanding of the potential clinical applications of these models. Finally, it's noteworthy that this study utilized pathologic features extracted by deep learning models. However, deep features inherently possess a “black box” nature, making it challenging to interpret the extracted characteristics.

In conclusion, building AI pathology models using multimodal data and clinical variables to predict the pathological staging of colorectal cancer is a crucial step toward more accurate and personalized healthcare. This study demonstrates the superiority of the combined model and nomogram, potentially providing diagnostic physicians with more information to make wiser staging assessments, thus enhancing patient management and treatment outcomes. Immunohistochemical data, such as Ki67, holds immense potential in the field of pathology and offers new directions for future pathological research and clinical applications.

## AUTHOR CONTRIBUTIONS

**Yang Tan:** Conceptualization (lead); data curation (lead); investigation (lead); visualization (supporting); writing – original draft (lead). **Run Liu:** Conceptualization (equal); data curation (equal); validation (equal); visualization (lead). **Jia‐wen Xue:** Resources (lead); software (lead); validation (lead). **Zhenbo Feng:** Methodology (lead); writing – review and editing (lead).

## CONFLICT OF INTEREST STATEMENT

The authors declare no conflict of interest.

## ETHICS STATEMENT

Ethical approval for this study was obtained from the Medical Ethics Committee of the First Affiliated Hospital of Guangxi Medical University. The approval notice, with reference number 2023‐E398‐01. The research protocol underwent a thorough review and was approved by the institutional Ethics Committee to ensure compliance with ethical standards and protection of the rights and interests of the study participants. This study is classified as a retrospective study, and the Ethics Committee has granted a waiver for informed consent. This statement is hereby declared.

## Data Availability

The data that support the findings of this study are available from the corresponding author upon reasonable request.
